# The Life and Times of Stephen B. McMahon (1954–2021)

**DOI:** 10.3389/fpain.2022.913232

**Published:** 2022-05-19

**Authors:** Anthony H. Dickenson, David L. H. Bennett

**Affiliations:** ^1^Department of Neuroscience, Physiology and Pharmacology, University College London, London, United Kingdom; ^2^Nuffield Department of Clinical Neurosciences, University of Oxford, Oxford, United Kingdom

**Keywords:** pain, McMahon, life and times, obituary, spinal injury

Steve McMahon, always known as Mac, was a remarkable scientist with a breadth of knowledge that spanned many areas of neuroscience and physiology but with a major emphasis on pain and spinal cord injury. Many scientists use a number of techniques in their career but Mac had a range that spanned *in vivo* and *in vitro* electrophysiology, immunohistochemistry, behavior, human psychophysics, pharmacology and molecular biology, all carried out to the highest standards. This range and his prolific output lead to an h index of 113 at the time of writing. His studies were innovative, ground breaking and always interesting. Mac trained a whole generation of young scientists, was a wonderful mentor and collaborator encouraging all who were around him. Attending a conference with Mac was a delight. His questions were always incisive but kind; the evening socializing was engaging but no one explained how he could be the last to leave but always on time for the morning session. In workshops and at advisory boards he was always on the ball and his scientific knowledge and wisdom were dispensed freely and with honesty. Mac loved to debate; such discourse was handled in a sympathetic fashion and although he was normally right he was prepared to change his position should the data or force of argument be persuasive. Mac had profound effects on the science of pain and spinal cord injury through his scientific output and wisdom for over 30 years and his legacy will live on.

Mac, a Londoner through and through, did an undergraduate degree in Physiology in Leeds in 1973 and stayed on to gain a Ph.D. in visceral sensory processing. In 1981, he moved back to London and joined the group of Pat Wall at University College London. He continued to work with Pat over many years and Pat moved his laboratory to join Mac when he left UCL as Emeritus. By this time Mac had set up his own research group from 1984 at St Thomas' Hospital Medical School and in 1996 became the Sherrington Prof of Physiology at Kings College London where he remained for the rest of his career. Pat and Mac both loved *in vivo* electrophysiology and brought their scientific expertise together in a series of imaginative experiments to understand how peripheral inputs and targets modulate the spinal processing of nociceptive inputs.

Mac worked on a wide range of topics with spinal cord repair and pain processing being the main themes. Mac loved to innovate and as his career flourished added more cutting-edge techniques to his group, enabling him to tackle scientific problems in a multi-disciplinary manner. He was instrumental in paving the way for anti-NGF therapies from a whole series of studies on the roles of neurotrophins in the sensory nervous system; the proof of concept for the analgesic actions of anti-NGF was ultimately validated by human trials. Unfortunately, side-effects on joint function have prevented their clinical use. His work became more and more translational. From studying inflammatory mediators in rat and human skin to combining neuronal activity in rodents to human psychophysical measures in humans, remarkable similarities were noted. And in one of his final studies, the paper was able to shed much needed light on the mechanisms behind neuromodulation for pain control.

Mac was a wonderful collaborator – the Dickenson and McMahon laboratories published 9 papers together where we had an idea and needed to marshal different techniques to succeed. Off we went – Ph.D. students and post-docs went from lab to lab and the studies raced ahead. Never a conflict or worry and the studies ranged from downstream mediators to cancer pain mechanisms to human sensory processing.

But the highlight of the collaborations was the establishment of the London Pain Consortium that went global and became the Wellcome Trust Pain Consortium. It all started when the Trust recognized the need for studies in systems for harnessing molecular and genetic advances in pain. Mac seized the day and a one-off 5 year Integrative Physiology Initiative Award was gained with eight PIs in London institutions. This was followed by two prestigious Strategic Awards and a Ph.D. program. For more than 15 years, collaborations were struck and the final personnel involved were Mac, ourselves, Irene Tracey, Allan Basbaum, Andrew Todd, Giandomenico Iannetti and Frank Porreca was a collaborator. A group of friends who all respected each other and with a thirst for scientific progress with Mac leading the team. The Consortium published innumerable papers, ran summer schools in the UK, Spain and Denmark, organized seminars, and the Ph.D. program produced a generation of excellent young scientists. The alumni of the Wellcome pain consortium are now establishing their own pain laboratories across the world.

Mac managed to integrate his scientific friendships with family life both with gatherings at his home in Muswell Hill London and where possible traveling with family. He is survived by his wife Sara, an academic pediatrician, daughter Emma and sons Arun and Jasso. Mac was such a respected scientist, a trailblazer, a wonderful friend and colleague and a delight to be with. He leaves a strong scientific legacy of scientific publications and the many people he has trained and inspired in the pain and neuroscience communities.

## Author Contributions

Both authors listed have made a substantial, direct, and intellectual contribution to the work and approved it for publication.

## Conflict of Interest

The authors declare that the research was conducted in the absence of any commercial or financial relationships that could be construed as a potential conflict of interest.

## Publisher's Note

All claims expressed in this article are solely those of the authors and do not necessarily represent those of their affiliated organizations, or those of the publisher, the editors and the reviewers. Any product that may be evaluated in this article, or claim that may be made by its manufacturer, is not guaranteed or endorsed by the publisher.

